# Proteomics and Epidemiological Models of Human Aging

**DOI:** 10.3389/fphys.2021.674013

**Published:** 2021-05-31

**Authors:** Ceereena Ubaida-Mohien, Ruin Moaddel, Ann Zenobia Moore, Pei-Lun Kuo, Faraz Faghri, Ravi Tharakan, Toshiko Tanaka, Mike A. Nalls, Luigi Ferrucci

**Affiliations:** ^1^Biomedical Research Center, National Institute on Aging, National Institute of Health, Baltimore, MD, United States; ^2^Center for Alzheimer’s and Related Dementias, National Institute on Aging, Bethesda, MD, United States; ^3^Molecular Genetics Section, Laboratory of Neurogenetics, National Institute on Aging, National Institutes of Health, Bethesda, MD, United States; ^4^Data Tecnica International, Glen Echo, MD, United States

**Keywords:** aging models, proteomics, biological pathways, epidemiological models, longitudinal cross-sectional, phenotypes, resilience, machine learning and artificial intelligence

## Abstract

Human aging is associated with a decline of physical and cognitive function and high susceptibility to chronic diseases, which is influenced by genetics, epigenetics, environmental, and socio-economic status. In order to identify the factors that modulate the aging process, established measures of aging mechanisms are required, that are both robust and feasible in humans. It is also necessary to connect these measures to the phenotypes of aging and their functional consequences. In this review, we focus on how this has been addressed from an epidemiologic perspective using proteomics. The key aspects of epidemiological models of aging can be incorporated into proteomics and other omics which can provide critical detailed information on the molecular and biological processes that change with age, thus unveiling underlying mechanisms that drive multiple chronic conditions and frailty, and ideally facilitating the identification of new effective approaches for prevention and treatment.

## Introduction

Understanding the aging process has been an important goal for humans since ancient history. However, only recently, biomedical research started considering aging as causally related to most chronic diseases and decline of physical and cognitive function (Sierra, 2016; Kaeberlein, 2017; Caballero Mora and Rodriguez Mañas, 2018). Recent evidence suggests that the rate of biological aging can be modified and that slowing down aging can substantially improve individuals’ health over their life-span. For example, studies in animal models demonstrated that longevity and health-span can be modified by genetic manipulation and pharmacological interventions. As these new concepts are acknowledged, and the proportion of the elderly population increases worldwide, the need to understand the biology of aging has taken center stage in the medical field and translated into the geroscience paradigm (Sanders et al., 2012; Sierra, 2016). Geroscience is an interdisciplinary field that seeks to define the biological mechanisms by which the aging process causes age-related diseases and disorders (Kaeberlein, 2017). Prevention of chronic disease based on the control of risk factors has led to the progressive reduction of morbidity and mortality over the past 50 years. However, with increasing frequency of multimorbidity in the population, prevention focused on specific diseases is unlikely to produce substantial improvements in population health. In fact, even if single diseases could be cured, the development of other age-related diseases in susceptible individuals would minimize its advantages (Kaeberlein, 2017). As an alternative approach, geroscience shifts the focus toward assessing the rate of aging as the driving risk factor for chronic disease and proposes that only slowing down aging can substantially improve both life-span and health-span (Sierra, 2016; Kaeberlein, 2017).

The operational translation of the geroscience paradigm requires the development of technology to assess aging’s biological underpinnings (Ferrucci et al., 2018, 2020). We hypothesize the existence of a network that encompasses many homeostatic mechanisms at molecular and cellular levels, in which the interplay and progressive imbalance between damage accumulation and effectiveness of the maintenance machinery determine the pace of aging and, over the long term, drive the emergence of the phenotypes and functional declines typical of “normal aging,” such as wrinkling of the skin or the decline in lean body mass. When this process is accelerated by environmental factors or coupled to specific genetic susceptibility, the severity of damage emerges clinically as diseases or geriatric syndromes. Slowing down this chain of events as soon as possible would have enormous clinical potentials to enhance health and expand health-span. However, research is needed to establish measures of aging mechanisms that are both robust and feasible in humans as well as to connect these measures to the phenotypes of aging and their functional consequences. In this review, particular focus will be given to how this has been addressed from an epidemiologic perspective using proteomics.

As a premise to such a discussion, it is important to understand how the field of aging research has evolved over the last decades. The study of biomarkers in medicine and geriatrics emerged during the 1970s (Sacher and Trucco, 1962; Woodbury, 1977) as an attempt to find tools to test hypotheses about the underlying mechanisms of aging. For example, Woodbury and colleagues proposed a random-walk model to understand the aging process, incorporating the longitudinal trends of biomarkers relevant to mortality (Woodbury, 1977). Vaupel et al. (1979) proposed the frailty model to address the issue of population heterogeneity. In this kind of frailty model, statisticians translate the heterogeneity by specifying multivariate failure time conditional on an unobserved construct, frailty, which can be both group and subject-specific. Other authors proposed that frailty is characterized by increased individual vulnerability to endogenous and exogenous stressors from aging-associated decline in reserve and function across multiple physiological systems (Xue, 2011; Buta et al., 2016; Takeda et al., 2020). The concept of frailty later evolved from a statistical construct to a clinical entity, and several operational definitions were proposed in the literature and generated a robust discussion in the field. The two most used sets of frailty criteria are the Fried’s Frailty Phenotype (Fried et al., 2001) and Rockwood and Mitnitski’s Frailty Index of cumulative deficits (Mitnitski et al., 2001; Caballero Mora and Rodriguez Mañas, 2018). The Fried frailty model is based on a pre-hypothesized cycle of physical frailty, which covers energy expenditure and intake, change in muscle mass and quality, decreasing resting metabolic rate, as well as physical functions and is quantified based on five criteria that may be measured in a clinical setting. Rockwood and Mitnitski proposed to quantify frailty as an accumulation of deficits (Caballero Mora and Rodriguez Mañas, 2018), a model that can be easily implemented for both research and clinical purposes. A shift in the central framework of aging research occurred in 2013, when two important papers hypothesized that the failure of a limited number of biological mechanisms are responsible for aging and sparked great enthusiasm in the field (Lopez-Otin et al., 2013; Kennedy et al., 2014). However, measures of these mechanisms are still in development and without standardized metrics, it is difficult to ascertain whether dysfunction of these mechanisms predicts clinical parameters of aging, such as multimorbidity or disability. The outcome definition of this research is also problematic, as it fails to capture the complexity of clinical presentations in aged individuals. Beyond inflammation and obesity, no risk factor for multimorbidity development has been identified to date. With an improvement in high-throughput -omics technology, including transcriptomics, proteomics and metabolomics, can provide critical detailed studies on the molecular and biological processes that change with age, unveiling underlying mechanisms that drive multiple chronic conditions and frailty, and ideally facilitating the identification of the new effective approaches for prevention and treatment (da Costa et al., 2016; Takeda et al., 2020).

## Epidemiological Models of Aging Research

### Comparison Between Cross-Sectional and Longitudinal Models of Aging

As the cost of “-omics” becomes increasingly affordable, their use in large epidemiological studies becomes very common. However, adaptation of epidemiological designs to this new technology is still a work in progress. For studies focused on the biology of aging, where questions are often centered on differences that occur across the life course, a key choice is a decision between cross-sectional and longitudinal designs and both models present challenges.

#### Cross-Sectional Designs

**FIGURE 1:**
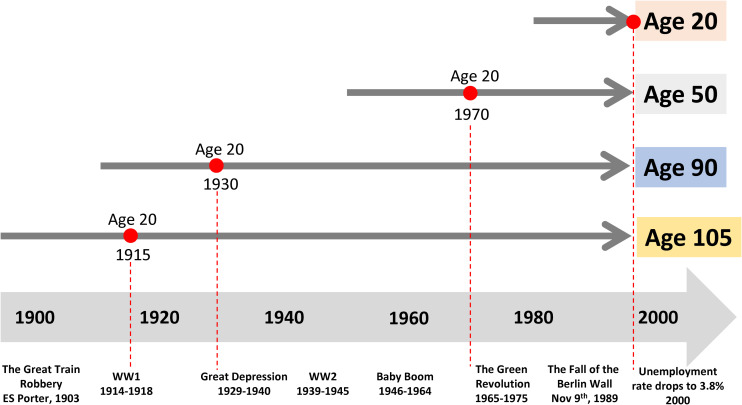
Past exposure to different environments affects the trajectories of aging and may bias the interpretation of cross-sectional studies. Let us assume that an investigator wants to compare individuals of different ages, such as 20, 50, 90, and 105. These individuals were 20 years old in very different historical epochs, which are as far apart as 85 years, characterized by extremely diverse access to food, stress, pollution and many other characteristics. Here, age- differences are indistinguishable from differences due to very different historical exposures.

In cross-sectional studies, participants are assessed at a single timepoint where time can be defined as a single calendar time frame or specified chronological age cohort. Cross-sectional studies are often nested in existing longitudinal cohorts. For example, Tanaka et al. (2020a) studied plasma protein levels in samples of participants evaluated at baseline (1998-2000) in the “Invecchiare in Chianti” (Aging in the Chianti Area, InCHIANTI) study, a community-based cohort study that has been followed longitudinally over more than two decades. Generally, cross-sectional studies can be performed relatively quickly on large populations. In the field of aging research, cross-sectional studies are often the only approach to compare individuals dispersed over a wide age-range, but these studies are vulnerable to biases that may be less frequently observed with other designs. The most important problem is distinguishing the effect of aging from secular trends. For example, let us assume that an investigator wants to compare individuals of different ages, such as 20, 50, 90, and 105 years old (Figure 1). These individuals were 20 years old in very different epochs, up to 85 years apart, where the early adulthood was characterized by extremely diverse access to food, stress, pollution and many other characteristics. Thus, having a different age also means different environmental challenges. At the extreme of this concept, the 105 years old participants may have more disease than the 50 years old not because of the age difference, but because at the age of 50 they had nutritional deprivation that is not present in the current 50 years old participant. Ergo, in cross-sectional studies, age differences are indistinguishable from differences due to secular trends. A historical example is the study that compared adults who were *in utero* during the Dutch Hunger Winter to controls born in other years. There was a clear and significant effect of the intrauterine environment on outcomes in later life, with higher likelihood of a metabolic syndrome phenotypes (Tobi et al., 2018).

**FIGURE 2:**
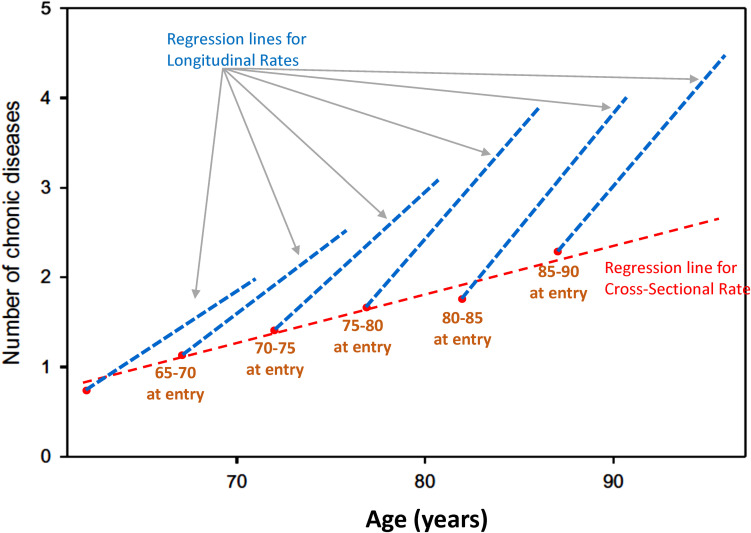
Age effects from cross-sectional studies are affected by selective attrition or loss to follow-up. Data from the BLSA show the relationship between age and multimorbidity. Estimates from longitudinal data in each cohort are much steeper than estimates obtained from baseline data only. In general, because mortality is more likely to occur at older ages, correlations with age may be attenuated because participants whose values are at the extreme of a distribution disappear from the population, and their hypothetical data are not observed (Figure 1).

**FIGURE 3:**
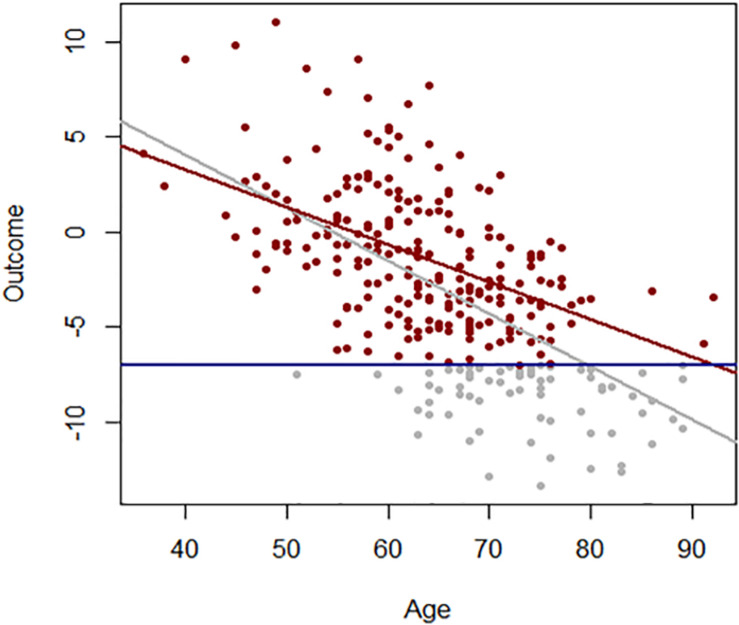
Association between age and a hypothetical outcome when all values of the outcome are observed (gray) and when values below a threshold (blue line) more frequently exceeded at older ages are not observed (red).

Age effects from cross-sectional studies are also affected by selective attrition or loss to follow-up. An example is illustrated in Figure 2 using data from the Baltimore Longitudinal Study of Aging (BLSA). The rate of change in multimorbidity with age estimated from longitudinal data is much steeper than what can be estimated using baseline data. In general, because mortality is more likely to occur at older ages, correlations with age may be attenuated because participants whose values are both within one extreme of distribution and consistent with earlier mortality are unobserved (Figure 3). In spite of these limitations, cross-sectional studies are frequently used, and as long as their limitations are accounted for, they are useful tools in the study of aging.

#### Longitudinal Designs

In contrast to cross-sectional studies, in longitudinal studies, participants are assessed repeatedly. Ideally, observations are equally spaced, but different time dimensions and metrics have been used and can be handled by statistical methods. High frequency of follow-up and long observation are often desirable in order to capture meaningful change in the dimensions of interest but can be cost-prohibitive and may increase the risk of loss to follow-up. In longitudinal studies of human aging, the time axis and nature of follow up are strongly influenced by life-span, physiologic events (e.g., menopause), disease processes, and social or behavioral milestones such as retirement. In the BLSA, a continuously enrolled study of community-dwelling adults designed to study the aging process, the period between study visits is dependent on the age of the participant: individuals <60 years old are seen every 4 years, participants 60-79 years are observed every 2 years, and participants ≥80 years of age, a period when rapid declines in health may occur, are followed up annually (Kuo et al., 2020). Because longitudinal studies are costly, labor-intensive, and the time required to produce results is long, investigators often introduce new measures within ongoing studies or use stored specimens for biological variables that can be correlated with phenotypic and outcomes information already collected. Identifying nested subsets of participants with appropriate follow-up patterns may be difficult, and length of storage may introduce a systematic bias in sample quality. These problems should be carefully evaluated in pilot studies, and to some extent, their effect can be blunted by appropriate statistical adjustment. Overall, longitudinal studies should be considered the tool of choice for studying aging.

### Modeling Healthy Aging as Compared to Normal Aging

Research into the biological mechanism of aging has both expanded and transformed. As mentioned above, the geroscience paradigm postulates that most chronic diseases and impairment in older adults are connected to the biological mechanisms of aging and that interventions that target these mechanisms will prevent disease and increase health-span (Sierra, 2016). Inherent in this paradigm is the assumption that aging and disease mechanisms may be distinguished, and the biology of aging research has increasingly focused on a set of putative mechanisms of aging, described as “pillars” or “hallmarks” of aging. However, many aging mechanisms may also be observed in overt disease independent of age. For example, while differences in DNA methylation, an epigenetic change, have been closely linked to aging phenotypes, the epigenome is also dysregulated in cancer and other diseases (Cavalli and Heard, 2019). It is appealing to turn to “healthy” populations to isolate and fully characterize mechanisms of aging. To pursue this strategy, key questions must be answered. How should the term ‘healthy’ be operationalized? Is health for the purposes of a study defined by the absence of disability, the absence of disease, clinical laboratory values within the normal range, self-reported health, or meeting fitness and functional testing thresholds? Further, does a given definition of health have the same meaning in all age groups? It is plausible that definitions such as “disease free” might induce a selection bias, as described for a cross-sectional study above, for example, if younger individuals who will develop the disease later in life are compared with older participants who have remained disease-free over the life course. However, in the context of careful study design and rigorous selection criteria, the evaluation of biomarkers may reveal subtle and meaningful age-associated variability in a healthy population that would be undetected in study samples drawn from ‘normal’ aging populations where aging and disease are intertwined.

### Assessing and Modeling Health-Span: The Compression of Morbidity

As mentioned earlier in this review, at least theoretically, there are substantial advantages in targeting the process that causes increased susceptibility to diseases for therapeutic purposes instead of implementing prevention for specific diseases, one by one (Newman et al., 2020). Similarly, especially when working with the older population, it is useful to consider developing diseases and disability as part of the same process (Fabbri et al., 2015; Newman et al., 2020). Under these assumptions, we can hypothesize that longitudinal trajectories of health are characterized by continuous decline (Kuo et al., 2020). We can define “health expectancy” as the period of life before health deteriorates below a predefined threshold and “unhealthy expectancy” as the period of life between the end of health expectancy and death (Kuo et al., 2020; Newman et al., 2020). For some diseases, this definition is adequate. For example, in dementia, most patients evolve across progressively more severe levels of cognitive impairment, and the reversal from this condition is a relatively rare (e.g., dementia due to normal pressure hydrocephalus that can be partially reversed by appropriate treatment) (Borelli et al., 2020). The situation is more complex for other diseases: many individuals undergo health status fluctuation, which becomes broader and more frequent in the last portion of life (Nguyen et al., 2020). Because of such cross-sectional and longitudinal heterogeneity, no satisfactory health-span measure is currently available, making it difficult to identify factors, such as diet or exercise, associated with better health trajectories over the life-span. At the population level and in a life-course perspective, the ratio between healthy and unhealthy expectancy is the best metric of health (Ferrucci et al., 2018; Liu et al., 2018; Kuo et al., 2020). The ideal outcome is to push health expectancy as close to life expectancy as possible, a strategy known by gerontologists as the “compression of morbidity.”

**FIGURE 4:**
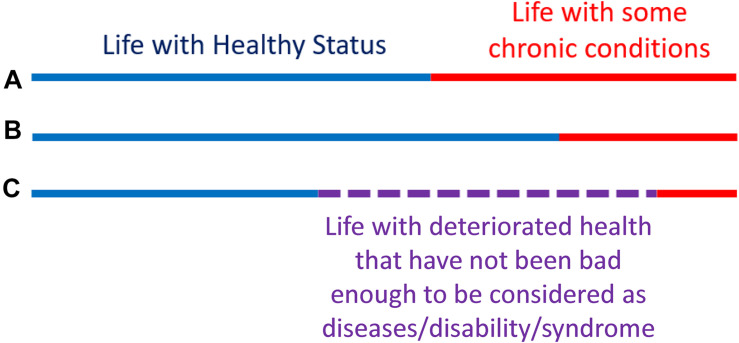
Health-span and life-span. The concept of “health-span” schematically divides life-span into “healthy” and a period of life characterized by chronic conditions and/or physical and cognitive impairment **(A,B)**. Although this rigid classification is attractive, it fails to recognize that trajectories of health are substantially more complex and periods of deteriorating health and function are interlaced with a period of recovery and relative well-being **(C)**. Thus, while a definition of health-span would be very useful, it has been difficult to operationalize, and no widely recognized definition is available at this stage.

**FIGURE 5:**
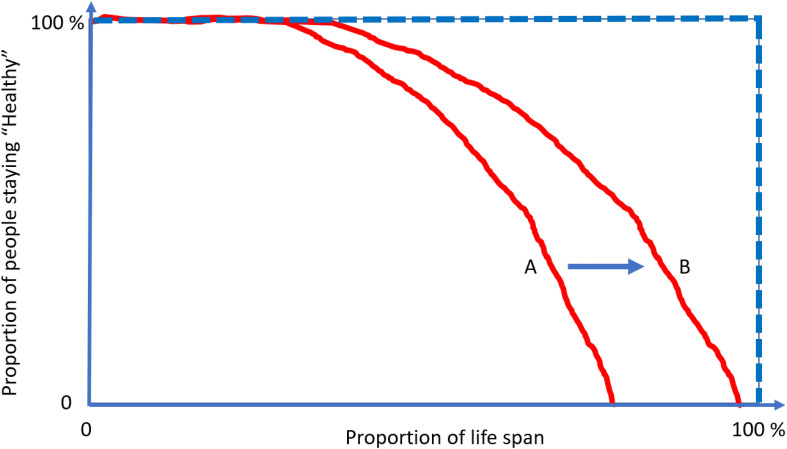
The compression of morbidity is the “Holy Grail” of gerontologists. Interventions that increase health-span disproportionally more in life span and compress the decline in health function toward the end of life. Current demographic and epidemiological data show no evidence for “compression” of morbidity but rather suggest that the period of life characterized by illness and disability is growing at a rate faster than healthy life. Focusing on the treatment of specific diseases when they become clinically manifest rather than on the promotion of health and resiliency cannot address this situation.

To better illustrate this concept, let us look at Figure 4. The lines represent the health trajectories of individuals A, B, and C. A and B have the same life-span, but individual B has a long health-span because the adverse health conditions happen late in life. The extension of this concept of health-span at the population level is shown in Figure 5. The survival curve A is for a population of individuals that develop health problems earlier in life and the survival curve B is for a population that stays healthy until near the end of life. Suppose there is an effective strategy for slowing down aging, the curve would move from A toward B. The complexity of defining health-span is that the definition of health is often not simply the presence or absence of disease. As presented by the life-span of individual C (Figure 4), people can often experience measurable deterioration in health, but maintain enough function to carry out living independently.

Meanwhile, the area above the curve, indicating the portion of people suffering from multiple conditions, would then decrease and realize the “compression of morbidity.” Using at least two age-related diseases as end points of health-span, we have demonstrated that higher plasma C-reactive protein (CRP) level is associated with 14% shorter health span for those who are older than 60 years old (Kuo et al., 2020). An extension from a specific protein, such as CRP, to discovery proteomics, will provide an indication of the mechanistic pathways that contribute to health-span. This unified concept of measuring global health has generated enthusiasm within epidemiology of aging, as witnessed by an exponential increase in the number of papers using health-span as an outcome. However, unlike life-span, which can be precisely defined as the age of death, capturing health-span within the boundaries of a rigid definition and in the context of global health have proved challenging (Newman et al., 2020). The emergence of a disease may be followed by the disappearance of signs and symptoms and a relatively long period of quiescence, such as some rheumatological diseases (Ellingwood et al., 2019). Also, predisposition to one disease may have a strong genetic component, and individuals may be subject to particularly powerful or prolonged environmental exposure (Rohn, 2014; Belloy et al., 2019).

An important consideration is that the focus on the phenotype of diseases and damage accumulation does not address the mechanisms of resilience, repair, and compensation (Ferrucci et al., 2020). An expanded use of biomarkers in medicine may potentially capture all aspects of the process by identifying biomarkers that track loss of resilience and predict health deterioration and its functional consequences. As discussed herein, measuring circulating proteins is particularly advantageous in this context because proteins are terminal effectors that can be linked directly to biological function (Ahmad et al., 2018; Tanaka et al., 2020b). Proteomics analysis in longitudinal studies in conjunction with clinical measures of health and functional changes may reveal patterns and signatures predictive of future changes in health and open an early window to the biological processes that are failing, therefore allowing precise and personalized interventions.

### Heterogeneity in Aging

Most research on aging deals with parameters that either decline (e.g., muscle strength, hearing) or increase (e.g., inflammatory markers, systolic blood pressure) with aging. The focus on “average” fails to recognize the fact that the individuals in the population become increasingly diverse or “heterogeneous” with aging. Age-dependent changes in heterogeneity are often conducted by comparing group means between young and old individuals. While group averages are informative, another metric of heterogeneity that is often overlooked is variance or the spread of a trait across age groups. The conventional wisdom dictates that for most traits, variance increases with age (Nelson and Dannefer, 1992). This idea is intuitive, that as individuals get older, there is an increased diversity as people are exposed to different environments and experiences and mount resilience responses to challenges that are even more variable. A recent study systematically examined the age-associated variance across thirty-four health characteristics representing eight domains (physical measures, vital signs, physiological measures, physical performance, function/disability, chronic conditions, frailty, laboratory values) (Nguyen et al., 2020). Interestingly, it was found that variance increased with age overall, but decreased (e.g., visual acuity, grip strength, Alanine Aminotransferase) or remained unchanged (CRP, gait speed, diastolic blood pressure) across age for some characteristics. Another interesting observation was that across all traits, the change in variance with age was non-linear, with peak heterogeneity observed at age 70. The increased variance in aging is also observed at the molecular level, where studies have shown increase in the variance of gene expression with age (Somel et al., 2006; Vinuela et al., 2018).

**FIGURE 6:**
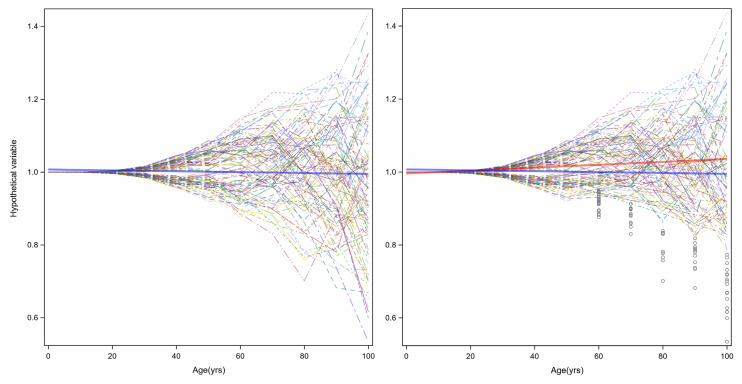
Age-related increase of heterogeneity of traits associated with health status may cause changes in average levels of that trait with aging. In the left panel, a hypothetical variable is perfectly controlled over the first years of life, but slowly and progressively, the homeostatic control becomes less effective, and variability increases symmetrically around the average (blue line). However, if the hypothetical variable is a “protective factor,” such as HDL-Cholesterol, and a person with low values has higher risk of cardiovascular disease, the consequent unobserved values show grey circles in the right panel the longitudinal distribution of age-trajectories truncated and asymmetrical. A linear regression with age (red) shows an increasing level.

The increased variance in gene expression can be described as stochastic dysregulation of gene expression resulting from the accumulation of damage over time. Age-related increase of heterogeneity of traits associated with changes in health status may explain the changing average level of that trait with aging. To explain this concept, Figure 6 displays simulation results of a certain hypothetical variable is perfectly controlled over the first years of life, but slowly and progressively, the homeostatic control becomes less effective, thus increasing variability in this case, symmetrically around the average (Figure 6, left panel). Now, let us assume that this hypothetical variable is a “protective factor,” such as high density lipoprotein (HDL) cholesterol, and individuals with low values have a higher risk of cardiovascular mortality or become sick and leave the study. The consequent unobserved values shown in gray circles in the right panel of Figure 6 make the longitudinal distribution of age-trajectories truncated and asymmetrical. A regression line (red) imposed on this data shows an increase in HDL levels with age. And since this variable increases with age, we may hypothesize that this factor is predictive of worsened health status, when in fact, the opposite is true. Hence, competing mortality and informative censoring cannot be ignored in the study of biomarkers of aging. Another important consideration is that variables that show increased heterogeneity are those with homeostatic controls that weaken with aging and whose changes are related to adverse age-related outcomes. Understanding the molecular and epidemiological bases of variance heterogeneity and how they relate to health-span is an important area that needs further development.

### Proteomics Models in Aging Research

The web of mechanisms underlying the maintenance of health is exceptionally sophisticated. A complex interaction of genetic, epigenetic, environmental and stochastic stresses continuously challenge such equilibrium and are offset by resilience strategies. The dynamic equilibrium between damage accumulation and repair may be detected by studying molecular changes in biological tissues and fluids. Of the different biomarkers, proteins are possibly the most interesting candidates because they are directly connected with function and because thousands of them can be measured in relatively small specimens. Recent studies suggest that the age-proteome is highly tissue-specific (Ori et al., 2015; Long, 2020), but few proteins change systematically with aging across selected tissues, such as the heart, lung and whole blood, suggesting a tighter co-aging pattern compared to other tissues such as muscle (Yang et al., 2015). Interestingly, non-dividing cells within a tissue may be more susceptible to changes due to extreme longevity, that agrees with an age-dependent decline of key regulatory proteins, as it has been observed (Arrojo et al., 2019). These long-lived proteins are more vulnerable to damage accumulation and function loss, and the study of their abundance may not reveal a strong aging signature.

### The Rationale for Using Proteomics in Building System Models in Humans

In the study of human aging, proteomics is often used as a tool for exploratory analyses or to address questions of reverse translation. In exploratory studies, the changes in protein composition of a different tissue with aging may suggest dysregulated biological pathways. In some instances, the findings in these studies are confirmatory; for example, a proteomic analysis performed on muscle biopsies from 58 participants in a study of the Genetic and Epigenetic Study of Aging Laboratory Testing (GESTALT) showed that both structural and functional mitochondrial proteins decline with aging (Ubaida-Mohien et al., 2019b). However, in the same study, we found a substantial overrepresentation of proteins from the splicing machinery with aging, a new and unexpected finding that led to a new hypothesis. More specifically, in the condition of a relative scarcity of energy, biological pathways are activated through alternative splicing that spare energy (i.e., inhibition of protein synthesis) and stimulate the production of energy (mitochondrial biogenesis, glycolysis). This hypothesis was verified by confirming that, after adjusting for potential confounders, splicing proteins were significantly associated with mitochondrial function assessed by P31 Magnetic Resonance Spectroscopy (Adelnia et al., 2020). A more recent approach to proteomics and aging is the development of “proteomic clocks.” Similar to what has been done with the methylome, elastic regression or other multivariate methods can be used to compute a weighted average of protein concentrations that approximate chronological aging and show deviation from chronological aging in individuals in a trajectory of deteriorating health (Lehallier et al., 2019; Tanaka et al., 2020a). Studies are ongoing to identify proteins that in asymptomatic individuals predict the development of phenotypes typical of aging, such dementia (Tanaka et al., 2020b), frailty (Landino et al., 2020) and many other diseases (Osawa et al., 2020; Tanaka et al., 2020b).

In spite of their potential and the already informative results, proteomic studies have limitations that need to be considered for accurate interpretation of their results. Indeed, before undertaking any substantial proteomics study, determinations of the instrument, sample, and human subject baseline variability must be made by preliminary experiments. These sources of variability must then be minimized by Sierra (2016) careful calibration of instrument parameters, (Kaeberlein, 2017) preparation of a pipeline for sample preparation that provides highly reproducible results, and possibly maximizes the use of robotics to reduce variability due to human errors, and (Caballero Mora and Rodriguez Mañas, 2018) identification of internal controls and standards such as reference samples and purified proteins and peptides. Similarly, occasionally running reference standards, or adding purified internal standards to proteomics runs, are often used in proteomics experiments to minimize and account for drifts in the measurement system or batch variability. Another important consideration is the abundance of the proteins of interest in a given tissue. If a target protein is of very low abundance, it may be very difficult to detect reliably by mass spectrometry. In these cases, some sample enrichment may be necessary, either by enriching the protein itself in the sample, for example, by an antibody enrichment, or by selecting specific cells in which the target protein is highly expressed. Furthermore, from their translation to their structural or signaling function, proteins undergo many changes that are only minimally captured by the current measurements. For example, correct folding, assemblage, post-translational modifications (PTMs) are essential for biological activity but require labor-intensive techniques to be assessed. Protein complex formation can be studied using so-called interactomics experiments, in which a protein of interest is ‘tagged’ with a label, allowed to be expressed in the cell, and then captured along with its interacting partners using an antibody against the tag. All of the interacting partners can then be identified using mass spectrometry (Burckstummer et al., 2006). These studies are usually performed in cell lines, but animal models may also be used. In human subjects, interactomics studies can also be performed using a ‘cross-linking’ approach, where a protein mixture is extracted in the native state, and treated with a cross-linking agent, such as formalin, to bind interacting partners tightly together (Gotze et al., 2019). The cross-linked sample can then be processed and the bound interactors identified. There are also specialized techniques by which post-translational modifications (PTMs) can be assessed. In these studies, a sample can be treated with an antibody specific to a PTM, which will select only proteins with the modification. Mass spectrometry can then identify all of the proteins while also confirming the PTM, since the mass shift is directly observable in the peptide sequence. Until recently, the study of human aging with proteomics methods was limited to small sample sizes (Staunton et al., 2012; Santos-Lozano et al., 2020). Unlike other “-omics” (genomics and transcriptomics) methods, the analytical efficiency of the traditional proteomics methods (LC MS/MS, 2Dgel, SRM) are time-consuming, expensive, and technically demanding. However, some proteome studies use the ‘sample multiplexing’ method, which takes advantage of pooling multiple isobarically labeled samples (TMT, iTRAQ) to increase throughput (Gygi et al., 1999; Ubaida-Mohien et al., 2019a). Recently developed proteomics technologies such as the aptamer-based arrays (SOMAlogic) or PEA multiplex technology (O-Link), and others, provide high-multiplexing of sample analysis but at the cost of a limited set of protein identifications. Despite the small sample size in proteomics studies and some intrinsic limitations of the methodology, proteomics studies of aging have already proven useful, especially when information on proteomics may be integrated with other -omics and with high quality phenotyping. For example, small sample size proteomics age model studies [44, 57, 58] and relatively big sample size studies [42, 59, 60] and large sample size studies [61-63] etc. often demonstrate the interplay of phenotypic-genotypic measurements in aging models. Importantly, both the quantity of sample available and the protein abundance in those samples may greatly limit proteomics analyses in terms of depth and reproducibility, and other laboratory techniques that are typically not high throughput should be used instead. For example, muscle aging and sarcopenia have been attributed to age-related degenerative processes in peripheral nerves and neuromuscular plaque. Material from neurons and neuromuscular plaque is just too small in any biopsy specimens to provide robust results by mass spectrometry analysis. Thus, although signals relate to denervation and neurogenesis are captured, these signals should be interpreted with caution. Similarly, stem cells may be very important for aging, but their numbers are so low that changes in their proteome are unlikely to be captured from a global muscle biopsy specimen. Recent development in low-input and even single-cell proteomics may begin to solve these problems, at some point in the future (Ctortecka and Mechtler, 2021).

**FIGURE 7:**
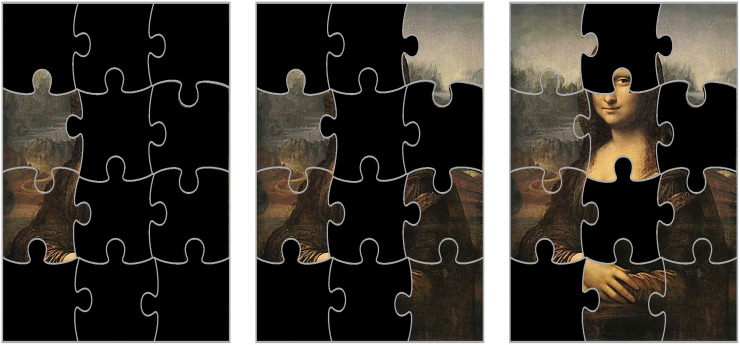
Because of the intrinsic limitation of proteomics, not all the proteins involved in a pathway are important for the mechanism studied and other proteins are detected but not fully quantifiable. Let us assume that biological pathways are puzzles, and each piece of the puzzle is a protein that belongs to that pathway. If we have most of the pieces of the puzzle (image on the right), the identification of the pathway is direct and simple: it is the famous Leonardo’s “Mona Lisa.” However, if we only have a few pieces available, recognizing the pathway may be complex (images center and left). In the study of aging, the situation is even more complex because many proteins are not unique but are rather promiscuous across pathways and our knowledge of these pathways (like the original paintings) is still limited.

A number of well-developed tools exist to identify proteins associated with specific traits, evaluate the strength of these associations, and graphically illustrate these relationships. However, similar to other “omics,” the biological and physiological interpretation of these findings remains problematic. Because of the intrinsic limitation of proteomics not all the proteins involved in biological pathway(s) are important for the mechanism studied or will be detected, and for others they are detected at low concentration. The signal-to-noise ratio may be too low to detect an association with the trait of interest. This results in an incomplete proteomic pictures of important biological pathways. Imagine that biological pathways are puzzles, and each piece of the puzzle is a protein that belongs to that pathway. If we have all the pieces of the puzzle, the identification of the pathway is direct and simple. However, if we only have a few pieces available, recognizing the pathway may be complex. The situation is complicated by the fact that most proteins are not unique but are rather promiscuous between pathways. Consider the images in Figure 7, the puzzle pieces that are available on the left image are insufficient to identify the painting underneath. However, as more pieces are matched, Leonardo’s “Mona Lisa” identity cannot be mistaken.

Similarly, while it is extremely arduous to make inferences from a list of proteins associated with a trait of interest considering them one by one, a specific set of proteins may contribute in concert to identify a biological process. “Gene set enrichment analyses” are methods to identify clusters of genes or proteins that are over-represented among those that are found associated with a trait of interest. Databases of these clusters are easily available in the public domain and were created by a comprehensive compilation of data available in the literature (Subramanian et al., 2005; Liberzon et al., 2015). Although every single published analysis on -omics report some form of ‘‘enrichment analysis,’’ this method has significant limitations. The genes or proteins included in a cluster are not ‘‘weighted’’ for importance or specificity. In addition, the quality of annotation is only as good as the available literature, which is biased toward certain fields, most notably cancer biology. This is particularly problematic for the field of aging research because age-related pathways have only recently been considered in annotation databases. Examples of using databases often used for enrichment analyses include the Kyoto Encyclopedia of Genes and Genomes (KEGG^1^) and Reactome (Jassal et al., 2020). These databases contain entries for all known human reaction pathways, including signaling pathways, metabolic pathways, and other cellular functions. The protein entries which are found to be aging-associated can be input into these databases, and the overlap between the input protein list and known pathways can be found. Some caution must be applied when interpreting the results of pathways analyses. First of all, important biological interactions may occur without substantial changes in protein concentration. For example, a pathway may include post-translational modification events among proteins, such as phosphorylation or ubiquitination cascades. Similarly, often the dysregulation of biological mechanisms with aging may be followed by both increase in abundance of certain proteins and a decline of abundance in other proteins within a pathway, making the interpretation of the results very difficult. Thus, the results of the pathways analysis must be manually curated in order to gain true biological information.

With the growing availability of proteomic and other “-omics” data in the public domain, it is now possible to correlate or compare a proteomics dataset with other datasets, either proteomics or other omics. Many public data repositories of published proteomics datasets exist such as PRIDE database (Schwenk et al., 2017; Perez-Riverol et al., 2019; Samaras et al., 2020), PeptideAtlas (Schwenk et al., 2017), and ProteomicsDB (Samaras et al., 2020) are all repositories of published proteomics datasets, and tools (Reisinger et al., 2015; Perez-Riverol et al., 2017, 2019) are available to mine them. A generated proteomics dataset can be correlated with public proteomics datasets from other tissues and other subjects and can also be correlated with non-proteomics data. For example, gene expression data such as RNA sequencing or other forms of microarray, stored in repositories such as Gene Expression Omnibus (Barrett et al., 2013), can be used to examine the relationship between the RNA level and protein level of various genes during aging. Proteomics data can also be annotated using various forms of metabolomics data, where a change in expression levels of an enzyme can result in a change in the abundance of the substrate/product of the enzyme and/or a downstream metabolite. Indeed, pathways analysis often much more useful when combined with metabolomic data, because metabolic pathways depend on the abundances of their components. Finally, genomic or epigenomic datasets may be used to annotate proteomics data. Large epigenomic repositories such as that of the ENCODE project (Davis et al., 2018) contain large amounts of epigenetic data, which can be used to correlate with the proteomic level. This genome-level data can be used to correlate protein-level data with subject genotypes.

### Advantages and Disadvantages Compared to Models That Use Other “-Omics” as Well as Phenotypic Data

Technological development of high-throughput assays for molecular biomarkers including genomics, epigenomics, transcriptomics, metabolomics and proteomics has lead to the rapid expansion of “-omics” research in aging. With the completion of the human genome project in 2001 (Venter et al., 2001), the first wave of -omics research on aging was conducted using genetic variants in genome-wide association studies (GWAS) (Melzer et al., 2020). While a handful of replicated genetic loci have been identified including ApoE and FOXO3A, large proportions of the variability in aging and aging traits remain unexplained by GWAS studies. Since inherited germline variants used in GWAS studies are static, there may be limitations to the capacity for genetic markers to capture the dynamic changes in aging. To this end, the dynamic nature of other -omics markers make them better candidates for biomarkers of aging and better tools to measure rates of aging that can be translated to the clinic. A promising line of research is developing epigenetic biomarkers where a series of aging indices were created using varying sets of DNA methylation sites, which were defined as “epigenetic clocks.” These epigenetic clocks have shown great promise as predictors of health and life-span (Levine et al., 2018). There is very little overlap in the set of CpGs used to construct the different clocks, however, evaluation of gene expression profiles suggests that the epigenetic clocks may be capturing genes in common molecular pathways such as metabolism, immunity and autophagy (Liu et al., 2020). However, the patterns of DNA methylation do not directly provide information on which genes are involved that make the epigenetic clocks powerful predictors of aging. In this respect, using proteomics in aging is advantageous as this approach directly measures the functional unit that affects phenotype. Identification of proteins that change with aging can provide direct insight into the molecular pathways that are influenced.

### Changes of Proteins With Aging: Biological Pathways Implicated and Current Models Underpinning the Mechanistic Association of Aging

The identification of biomarkers that can be integrated with health outcome has been a priority in aging research for the last 50 years. Prior to the -omics era, this area of research started by characterizing the relationship between a single biomarker with aging and age-related health outcomes. For example, IL-6, perhaps considered one of the first aging biomarkers, was described as “a cytokine for gerontologists” (Ershler, 1993). IL-6 is the best characterized biomarker for “inflammaging,” a chronic low-grade inflammation that develops with advanced age and contributes to the pathogenesis of age-related diseases and most recently has been recognized as a core element of the secretome produced by senescent cells (Lopez-Otin et al., 2013; Ferrucci and Fabbri, 2018; Franceschi et al., 2018). High circulating levels of proinflammatory cytokines, such as IL-6 have been associated both cross-sectionally and prospectively with major age-related chronic diseases as well as with disability and frailty (Maggio et al., 2013). The mechanisms of these associations are unclear and have been connected with down-regulation of the biological activity of Insulin-like Growth Factor-1 (IGF-1), which contributes to the decline of muscle strength with aging (Maggio et al., 2013; Johnson et al., 2020; Moaddel et al., 2021). A number of other inflammatory biomarkers have been associated with aging and age-associated health and functional deterioration, including C-Reactive Protein, Cystatin-C, TNF-alpha receptors I and II, Interleuking-1 receptor antagonists and many others (Dinarello, 2006; Jylha et al., 2007; Odden et al., 2010; Bauernfeind et al., 2016).

Most recently, scientists realized that information on individual protein levels is insufficient to understand the complex mechanisms of aging and chronic diseases and the focus shifted to protein “signatures” or a multi-protein model (Tanaka et al., 2018; Lehallier et al., 2019; Moaddel et al., 2021). In our recent study, a weighted average of 76 proteins were found that correlated with chronological age, and independent of chronological age predict health outcomes (Tanaka et al., 2018). Subsequent “proteomic” clocks developed in larger study populations and using an expanded set of measured proteins substantially confirmed the top-related proteins. Special mention should be given to Growth/differentiation factor 15 (GDF15) a member of the transforming growth factor−β cytokine superfamily that is strongly correlated with chronological aging even in healthy individuals, is a strong risk factor for cardiovascular disease, and strongly predict age-related adverse health outcomes, including obesity, cancer, neurodegenerative diseases, metabolic diseases, cognitive impairment and frailty (Wollert et al., 2017; Cardoso et al., 2018; Tanaka et al., 2018; Lehallier et al., 2019). The mechanisms by which GDF-15 is associated with these outcomes are unclear, but appear to be related to modulation of the inflammatory response and perhaps regulation of appetite and body composition.

Two recent systematic reviews have identified age-associated proteins assessed with different methods and with different matrixes (Johnson et al., 2020; Moaddel et al., 2021). When enrichment analysis was performed on the list of proteins identified in these reviews, the biological pathways that emerged are widely recognized as playing a role in aging (Johnson et al., 2020; Moaddel et al., 2021). These pathways include the human complement system, the IGF1 signaling pathway, the PI3K-Akt-mTOR-signaling pathway, the mitogen-activated protein kinases (MAPK) signaling pathway, the Hypoxia-inducible factor 1 (HIF-1) signaling pathway, cytokine signaling pathways, FOXO signaling pathway, Advance glycation end products (AGE)/receptor AGE (RAGE) pathway, folate metabolism and mRNA splicing-major pathway. The signaling pathways are not operating independently of each other but rather are interconnected with several pathways regulating or co-regulating each other. For example, IGF-1/AKT pathway co-regulates the mTOR pathway (Feng and Levine, 2010) and is involved in an array of different processes, including metabolism, cell proliferation, survival and synaptic plasticity (Feng and Levine, 2010; Mazucanti et al., 2015). IGF1 binds to the IGF1 receptor, leading to a conformational change resulting in the activation of receptor tyrosine kinase activity (Hakuno and Takahashi, 2018). Several substrates are phosphorylated, including insulin receptor substrates and Src homology collagen (SHC), which activate phosphatidylinositol 3-kinase (PI 3-K)-Akt signaling pathway and the Ras-mitogen-activated protein kinase (MAP kinase) pathway. The Insulin/IGF signaling pathway also triggers the phosphorylation of FOXO factors via AKT, resulting in the suppression of FOXO-dependent transcription of target genes (Floyd et al., 2007; Morris et al., 2015; Chen et al., 2020). mTORC1 inhibits autophagy and regulates mitochondrial function and glucose metabolism via HIF1α, a critical transcription factor which plays a role in aging-related pathology including chronic inflammation, cellular senescence and neoplastic development (Anisimov, 2015; Ebersole et al., 2018; Alique et al., 2020). HIF1 activation has been shown to be dependent on RAGE-mediating signaling (Semba et al., 2010). HIF1α downregulates mitochondrial biogenesis, thereby reducing ATP production, and potentially contributing to the oxidative stress that triggers the nuclear factor κB (NFkB)-mediated production of cytokines (Yeo, 2019). NF-κB a major transcription factor that is activated in many diseases associated with inflammation and aging, such as arthritis, atherosclerosis, diabetes, Alzheimer’s disease and Parkinson’s disease (Chen et al., 2020).

### Does Accelerated Aging Predict Adverse Health Outcomes?

**FIGURE 8:**
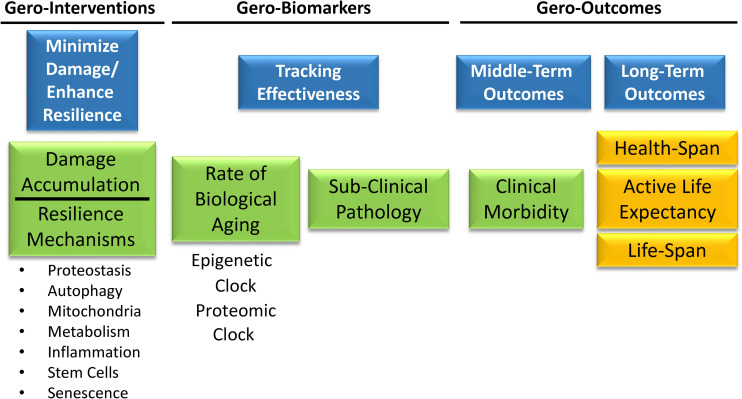
Gero-interventions aim to target one or more putative mechanisms of aging among those already discovered, or others still undiscovered. A primary aim will be demonstrating a beneficial effect on the rate of biological aging estimated by biomarkers signatures, such as the “epigenetic” or the “proteomic” clock, and on some subclinical pathology such as “arterial stiffness” or “amyloid accumulation.” Another aim will be to demonstrate that changes in biological aging and subclinical pathology prevent middle-term outcomes important for aging, such as multimorbidity or lower extremity performance. Finally, these trials will need to demonstrate that the intervention substantially changes the trajectory of aging by preventing physical and cognitive disability and promoting longevity.

A primary goal of biomarker discovery in the field of aging is measuring the rate of biological aging. In a translational perspective, measuring accelerated aging would be important to identify individuals who are ideal targets for interventions aimed at slowing down the aging process and prevent its consequences on health, functional status and quality of life. Measuring the rate of aging can be used to track the effects of behavioral interventions. For example, are changes in health behavior, such as smoking cessation or increase of physical activity, associated with changes in the rate of aging? A measure of the rate of aging can be used to track the effectiveness of interventions aimed at slowing down aging and therefore preventing its deleterious consequences on health and function without having to wait to directly observe health related outcomes such as disability or mortality. Ultimately, aggregate biomarkers of aging, such as the “epigenetic clock” or the proteomic clock will play an essential role in the design of a new generation of clinical trials (Figure 8). Geroscience-based interventions will target one or more putative mechanisms of aging, among those that are already discovered or some other still undiscovered. A primary aim will be demonstrating that these interventions have a beneficial effect on the rate of biological aging estimated by biomarkers signatures, such as the “epigenetic” or the “proteomic” clock, and on subclinical pathology such as “arterial stiffness” or “amyloid accumulation.” A second primary aim will be demonstrating that changes in biological aging and subclinical pathology prevents middle-term outcomes important for aging, such as multimorbidity or lower extremity performance. Finally, these trials will need to demonstrate that the intervention substantially changes the trajectory of aging by preventing physical and cognitive disability and promoting longevity. Substantial work in the field of aging research is taking place to develop the tools and identify these trials’ interventions.

## Conclusion and Future Perspective

### Future Framework for Machine Learning and Artificial Intelligence in Models of Aging

As we accumulated an unprecedented amount of biomarkers data, our ability to process and extract all the hidden data and information did not grow at a similar rate, and new analytical techniques are being developed to accomplish this goal. Machine Learning (ML) algorithms and techniques as part of a broad field of Artificial Intelligence (AI) have made significant strides and are widely used in many areas, including healthcare and biomedicine. Although sometimes researchers view these concepts as a panacea for all analytic challenges, one should be mindful of their limitations and practical constraints. Often times a conventional association study or exploration of descriptive statistics may be more situationally appropriate. Where ML/AI really shines is by making data-driven inferences that may not be readily observable using standard analysis, using ML methods and automated processes that learn and interact with humans or other computing AI. Within the context of biomedical aging research, we see a few key areas of growth in the adoption of ML/AI to accelerate scientific progress, especially developing therapeutic targets. Drugs with a genetic basis are almost twice as likely to pass a phase of a trial than those without (King et al., 2019). Many recent efforts in drug development pursue a network or pathway-based approach for target identification, although many pathways have implicit biases, particularly those from literature searches and text mining. Currently, a wealth of multi-omics data is being generated on an unprecedented scale. With methods such as network-based clustering, we can build communities of functionally and phenotypically related genes representing *de novo* networks and pathways. Drug developers can target nodes within these clusters for disruption by pharmaceuticals (Traag et al., 2019). Additionally, ML model interpretation can help prioritize interesting biomarkers and targets for future studies.

There are challenges ahead for ML/AI breakthroughs to achieve these goals and as much impact in the aging domain as in other healthcare areas. Some major challenges are: data diversity (training the data model with the same demographic diversity in the age, gender, and racial/ethnic composition of data), strong machine learning models (the models should be reproducible, usable, and interpretable for aging research), data privacy and ethics (deployment of machine learning models with ethical considerations), domain knowledge and global collaboration and data sharing. Global data sharing is crucial, considering the ethnicity and diversity complexities in aging studies; the only way to developing highly generalizable ML/AI solutions for aging research is global collaboration and data sharing. We have mentioned earlier the public domains and data repositories in proteomics research however, many aging data are still not available in the public domain. A public aging data deposition followed by an open science, code sharing, and public analytical documents allow independent replication of the results, and this will certainly lead to progress.

### Challenges Ahead for a Unified Model for the Integration of Biological, Phenotypic, and Functional Measures of Aging

**FIGURE 9:**
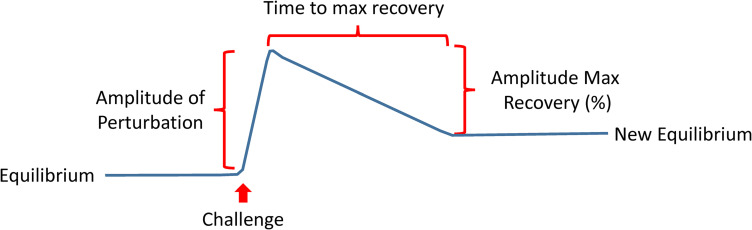
The development of a new generation of challenging tests that assess resilience is a priority in aging research. These tests may take different forms ideally but will be targeting mechanisms of aging, such as autophagy, proteostasis response, mitochondrial function and cellular senescence, but also new phenotypes such as response to changes in temperature or sustained fasting. Depending on the nature of the test, different parameters could be derived as indicated in the figure.

The recent shift of Geroscience paradigms to understand biological aging and age-related diseases at the molecular level gives far-reaching hopes that research on aging, from mere curiosity, will be a driving force of a new era of medicine. A recent review has described the hallmarks of aging as molecular events such as genomic instability, loss of proteostasis, deregulated nutrient sensing, mitochondrial dysfunction, cellular senescence, stem cell exhaustion, and altered intercellular communication (Lopez-Otin et al., 2013). Likely many more “hallmarks” will be discovered in the future, and their interconnection will be better defined, but the current list is a good starting point. Much work is needed to interconnect these mechanisms with phenotypic manifestations of aging, including diseases, multimorbidity as well as the decline of physical and cognitive function. A roadblock of this research is our limited ability to measure compensation, which is the result of centuries of medical research and practice that focused on measuring degree of damage and “disease severity” (Ferrucci et al., 2018). Indeed, if we want to measure resilience, we will need to expand the use of challenge tests in research and eventually also in clinical practice. If resiliency mechanisms are effective above a certain level, there will be no evident damage, and therefore, measuring damage will not be useful. Many challenge tests are already used in medicine, such as the oral glucose tolerance for the detection of latent diabetes, the treadmill stress test for the detection of coronary insufficiency, or the Dexamethasone suppression test for the diagnosis of the Cushing syndrome. New challenge tests that target mechanisms of aging, such as autophagy, proteostasis response, mitochondrial function and cellular senescence, but also new phenotypes such as response to changes in temperature or sustained fasting should be developed and their results correlated with clinical outcomes. Depending on the nature of the test, different parameters could be derived, such as the amplitude of the perturbation, the time to recovery, the percentage recovery compared to baseline (Figure 9).

**FIGURE 10:**
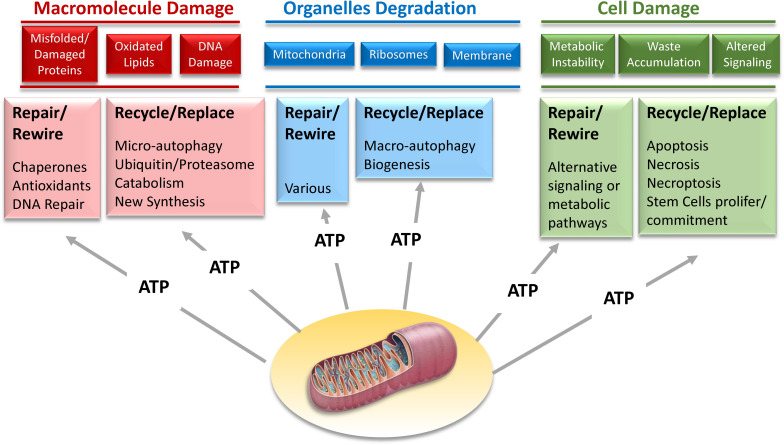
Mechanisms of resilience that maintain homeostatic stability on the dynamic network of life aim to repair damage at macromolecular, organelle, and cellular level. However, when the repair is not possible, recycling and replacing these macromolecules, organelles and cells is a suitable alternative. Most of these strategies of biological resilience requires large amounts of energy that can only be provided through mitochondria oxidative phosphorylation.

However, improvements in biomarker technology may open the door to a new strategy. There is initial evidence that the activation of resilience mechanisms produce a biomarker signature that can be detected in tissues or biological fluids. For example, it has been demonstrated that acute stress leads to the release in plasma of mitochondrial DNA (Hummel et al., 2018; Trumpff et al., 2019). Thus, studies that combine the results of challenge tests with -omics may be used to delineate those signatures that, once validated, can be translated into diagnostic tools in epidemiological studies and later on to the clinic. Since most resilience mechanisms require extra-energy, we believe that the secret of aging lies in the progressive derangement of energetic metabolism. Developing a new generation of challenge tests and resilience biomarker signatures, some of which are related to mitochondrial function and oxidative phosphorylation, the major source of energy (Figure 10), opens the road to studies looking that the effects of genetic and environmental factors of resilience capacity.

## Author Contributions

LF and CU-M designed the manuscript. LF supervised the writing of the manuscript. CU-M, RM, TT, AZM, P-LK, FF, RT, MAN, and LF wrote the paper.

## Conflict of Interest

FF and MAN participated in this study as a part of a contracting agreement between their company (Data Tecnica International, LLC) and the National Institutes of Health. The remaining authors declare that the research was conducted in the absence of any commercial or financial relationships that could be construed as a potential conflict of interest.

## Footnotes

1 https://www.genome.jp/kegg/pathway.html
